# The prognostic significance of human epidermal growth factor receptor family protein expression in operable pancreatic cancer: HER1–4 protein expression and prognosis in pancreatic cancer

**DOI:** 10.1186/s12885-016-2889-6

**Published:** 2016-11-21

**Authors:** Qin Li, Lei Zhang, XiuHong Li, Han Yan, Liuting Yang, Yingying Li, Teng Li, Jing Wang, Bangwei Cao

**Affiliations:** 1 Department of Oncology, Beijing Friendship Hospital, Capital Medical University, Beijing, 100050 China; 2 Department of Pathology, Beijing Friendship Hospital, Capital Medical University, Beijing, China; 3 Research Experiments Center, Beijing Friendship Hospital, Capital Medical University, Beijing, China; 4 Department of Biochemistry and Molecular Biology, Basic Medical College, Shanxi Medical University, Taiyuan, China; 5 Department of Pathology and Pathophysiology, Basic Medical College, Capital Medical University, Beijing, China; 6 Beijing Key Laboratory for Precancerous Lesion of Digestive Diseases, Beijing Friendship Hospital, Capital Medical University, Beijing, China

**Keywords:** Pancreatic cancer, Prognosis, HER1, HER2, HER3

## Abstract

**Background:**

Prognostic factors aid in the stratification and treatment of cancer. This study evaluated the prognostic significance of human epidermal growth factor receptor (HER) family members (HER1–4) expression in patients with operable pancreatic cancer.

**Methods:**

The expression of individual HER proteins in patient tissue specimens was detected by immunohistochemistry staining. Patient follow-up time was between 1.0 and 78.1 months.

**Results:**

Positive expression of HER1, HER2, HER3 and HER4 was detected in 41.4, 60.0, 24.3 and 65.7% of cases, respectively. Kaplan–Meier analysis revealed that HER3 positive expression was associated with decreased median survival time (12.0 vs. 25.6 months for HER3 positive and negative groups, respectively; *P* = 0.013). Cox’s regression confirmed that positive HER3 expression was an independent predictor of poor survival (RR = 3.684, *P* = 0.001). In contrast, HER4 negative patients had a significantly decreased median survival time when compared with HER4 positive patients (11.4 vs. 25.6 months, respectively; *P* = 0.027). However, HER4 was not an independent predictor of survival. No significant association between HER1 or HER2 expression and survival was observed (*P* = 0.626 & *P* = 0.859, respectively).

**Conclusions:**

HER3 is an independent prognostic marker for patients with operable pancreatic cancer. HER4 may also be of potential prognostic value in this disease and deserves further attention.

## Background

Pancreatic cancer is the fourth leading cause of cancer-related death in the United States with a 5-year survival rate of only 5% [1]. The majority of patients are diagnosed in the late stages of disease both because of a lack of effective early detection methods and a lack of recognizable symptoms. Although surgical resection offers the best hope for long-term survival [2], post-surgery relapse or metastasis are inevitable [3]. As a result, there is an urgent need to discover new biomarkers to aid in predicting prognosis and developing curative strategies against pancreatic cancer.

The human epidermal growth factor receptor (HER) family belongs to the tyrosine kinase receptor superfamily. It includes four highly homologous members, HER1, HER2, HER3 and HER4. HER family proteins and their associated downstream signaling networks regulate cellular activities such as gene expression, mitosis, differentiation, proliferation, survival, migration, invasion, and apoptosis [4, 5]. In the clinic, targeted therapy directed at HER family proteins is well established. The small molecule HER1 tyrosine kinase inhibitor gefitinib has been used in the fist-line treatment of advanced non-small cell lung cancer associated with HER1 mutation [6]. The anti-HER1 monoclonal antibody cetuximab has been used in the targeted therapy of head and neck squamous cell carcinoma, colorectal cancer and advanced non-small cell lung cancer [7–9]. The anti-HER2 monoclonal antibody trastuzumab has been used in the targeted therapy of HER2-positive advanced breast and gastric cancers [10, 11]. The bispecific antibody MM-111 and anti-HER3 antibody patritumab both inhibit the activation of HER3 and demonstrate antitumor activity [12, 13], while HER4 represents a potential molecular target for melanoma [14]. HER family proteins are also of important clinical significance in determining prognosis in a variety of solid tumors. HER1 overexpression is associated with increased depth of invasion, vascular invasion, and poor prognosis in esophagus squamous cell carcinoma [15]. HER2 overexpression is associated with shorter time to progression and decreased survival in breast cancer [16]. HER2 and HER3 are both predictors of poor outcome in gastric cancer [17, 18]. Data on the prognostic value of HER4 in solid tumors are less clear. However, a study by Barnes et al. demonstrated that absence of HER4 expression could predict the recurrence of ductal carcinoma in situ of the breast [19].

The prognostic significance of HER family protein expression in operable pancreatic cancer remains unclear. This study was to investigate the expression status of HER family proteins (HER1–4) in tissue specimens obtained from patients with operable pancreatic cancer. A detailed analysis of the relationship between HER family expression status and patient survival was performed, and factors that represented independent predictors of prognosis were identified.

## Methods

### Cell culture, antibodies and other reagents

The AsPC-1 human pancreatic cancer cell line was obtained from the experimental center of Beijing Friendship Hospital. Cells were cultured in Dulbecco’s Modified Eagle Medium supplemented with 10% fetal bovine serum and maintained at 37 °C in a humidified incubator containing 5% CO2.

For western blotting, rabbit monoclonal antibodies against phospho-HER3 (Y1289) and beta-actin were purchased from Cell Signaling Technology. The anti-HER3 monoclonal therapeutic antibody (clone 3D4) was obtained from Beijing Cotimes Biotech Co., Ltd. and used at 2.5 μl/ml for 0.5 h. The anti-human HER2 antibody (Herceptin) was purchased from Shanghai Roche Pharmaceuticals co., LTD and was used at 7ul/ml for 2.5 h.

### 3-(4,5-dimethylthiazol-2-yl)-2,5-diphenyltetrazolium bromide (MTT) assay

To perform MTT-based cell proliferation assays, cells in the logarithmic phase of growth were seeded in triplicate in 96-well plates at a density of 0.5 × 104 cells/well. Cells were then treated with serially diluted anti-HER3 monoclonal antibody (clone 3D4, Beijing Cotimes Biotech Co., Ltd.) for 48 h, after which 10 μl of a 5 mg/ml stock of MTT (Sigma) was added to the wells. The cells were incubated at 37 °C for an additional 4 h, and the reaction then stopped by treating the cells with 150 μl of DMSO for 5 min. The optical density was then measured at 490 nm. A control group that received no antibody treatment was used as a reference for calculating relative cell survival.

### Western blotting analysis

AsPC-1 human pancreatic cancer cell line were lysed, and cell lysates were resolved on 7.5% SDS–PAGE gels, and the proteins then electrophoretically transferred onto nitrocellulose membranes. Blotting membranes were blocked for 1 h and incubated with primary antibody overnight at 4 °C. Goat anti-β-actin was used as a loading control. Membranes were washed with Tris-buffered saline/0.5% Tween 20 and then incubated with anti-rabbit (for primary antibodies against HER family proteins) or goat (for the primary β-actin antibody) lgG secondary antibody conjugated to horseradish peroxidase. For protein visualization, nitrocellulose membranes were incubated for 2–3 mins with Western HRP Substrate and the chemiluminescent product then detected following exposure to KODAK film.

### Patients

Seventy patients who underwent surgical resection for primary pancreatic cancer at the Beijing Friendship Hospital between January 2008 and December 2012 were enrolled in this study. Complete clinical, pathological and follow-up data were obtained for all patients. Clinical data including age, sex, family tumor history, smoking and drinking history, and CA 19-9 score were recorded. Pathological data including tumor location, margins, histology grade, histopathology type, primary tumor (T), lymph nodes (N) and the number of positive lymph nodes were also collected. The median age of all patients was 62 years, with a range of 18–80 years. Follow-up time was between 1.0 and 78.1 months. The deadline of follow-up was December 31, 2015, or the data of patient death.

### Immunohistochemistry (IHC) and tissue scoring

Five-micron-thick sections were prepared from formalin-fixed, paraffin-embedded tissues derived from surgically resected pancreatic cancer specimens. Immunohistochemical samples were individually assessed without knowledge of patient outcome. Prior to staining, each slide was deparaffinized and heated in citrate buffer. After cooling and rinsing in Tris buffer, immunohistochemical staining was performed using an automated IHC autostainer.

The kits for the detection of HER family proteins (HER1–4) were purchased from Santa Cruz Biotechnology. Inc. Anti-HER1 antibody (sc-03, Rabbit polyclonal IgG), anti-HER2 antibody (sc-284, Rabbit polyclonal IgG), anti-HER3 antibody (sc-285, Rabbit polyclonal IgG), and anti-HER4 antibody (sc-283, Rabbit polyclonal IgG) were used at a dilution of 1:200, 1:200, 1:20, and 1:40, respectively. Detection of HER family proteins was performed using a secondary mouse anti-immunoglobulin antibody linked to biotin in conjunction with streptavidin linked to horseradish peroxidase.

Slides were scored independently by two individuals using a 4-point intensity scale system, and any discrepancies in scores were resolved by an arbiter. HER protein staining was scored as either 0, 1+, 2+, or 3+, the cytoplasm and membrane-staining intensity and pattern were evaluated according to the following scale [20]: no staining is observed or is observed in less than 10% of the tumor cells (score 0), weak staining is detected in 10% or more tumor cells (score 1+), moderate staining is observed in 10% or more tumor cells (score 2+) and strong staining is observed in 10% or more tumor cells (score 3). Scores of 0, 1+ and 2+ were taken to represent negative protein expression, while a score of was 3+ was taken to represent positive protein expression.

### Statistical analysis

Survival time was calculated in months from the date of surgery to the date of death, or date of final contact as of December 31, 2015. The survival times of patients between different subgroups were compared using the log-rank test. The Chi-square test was used to test associations between the expression of HER proteins and clinicopathological parameters. The Kaplan–Meier method was used for survival analysis and the log-rank test to determine the statistical significance of differences between survival curves. Relative risks associated with HER positive expression were calculated by Cox’s Multiple Regression analysis. A P value of less than 0.05 was considered significant. Statistical analysis was performed using SPSS 11.0 software (SPSS Inc., Chicago, IL, USA).

## Results

### HER3 expression in pancreatic cancer cells and the inhibitory effects of anti-HER3 antibody

**Fig. 1:**
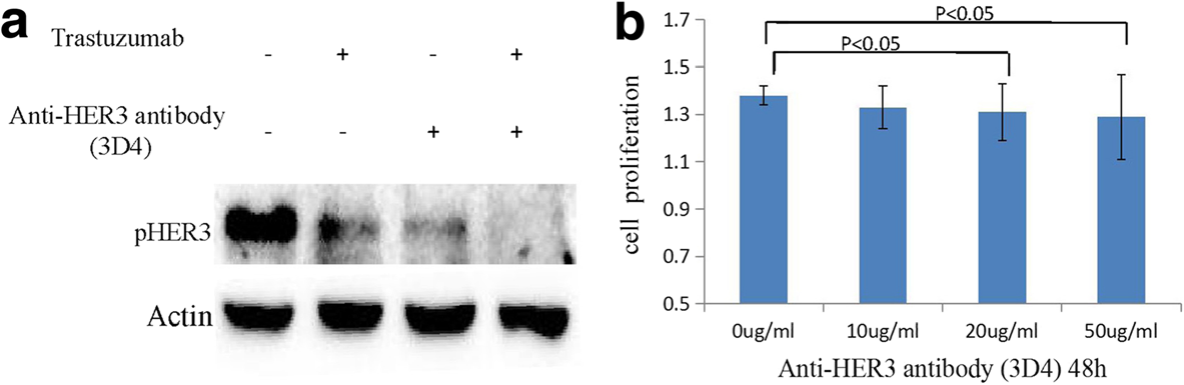
pHER3 expression in AsPC-1 cells and the inhibitory effect of anti-HER3 monoclonal antibody (3D4). **a**. pHER3 was overexpressed in the AsPC-1 cells. Both 3D4 and trastuzumab significantly down-regulated the expression of pHER3, and two drug combination showed synergistic inhibitory effect. **b**. 3D4 significantly inhibited cellular proliferation at concentrations equal to 20 ug/ml or 50 μg/ml (*P*  <  0.05)

Given the significant association observed between HER3 expression and poor survival in patients with many solid tumors, we wished to determine whether this receptor was expressed in pancreatic cancer cells in vitro and, if so, to determine its role in cellular proliferation. High levels of pHER3 expression were confirmed in the AsPC-1 pancreatic cancer cell line by western blotting, anti-HER3 monoclonal antibody (3D4) and anti-HER2 monoclonal antibody (trastuzumab) significantly down-regulated the expression of pHER3, and two drug combination showed synergistic inhibitory effect (Fig. 1a). Furthermore, the treatment of AsPC-1 cells with increasing concentrations of anti-HER3 monoclonal antibody (3D4) resulted in significant inhibition of cellular proliferation at concentrations equal to 20 ug/ml or 50 μg/ml (*P* < 0.05), as determined by an MTT assay (Fig. 1b).

### Expression of HER family proteins in pancreatic tissue specimens

**Fig. 2:**
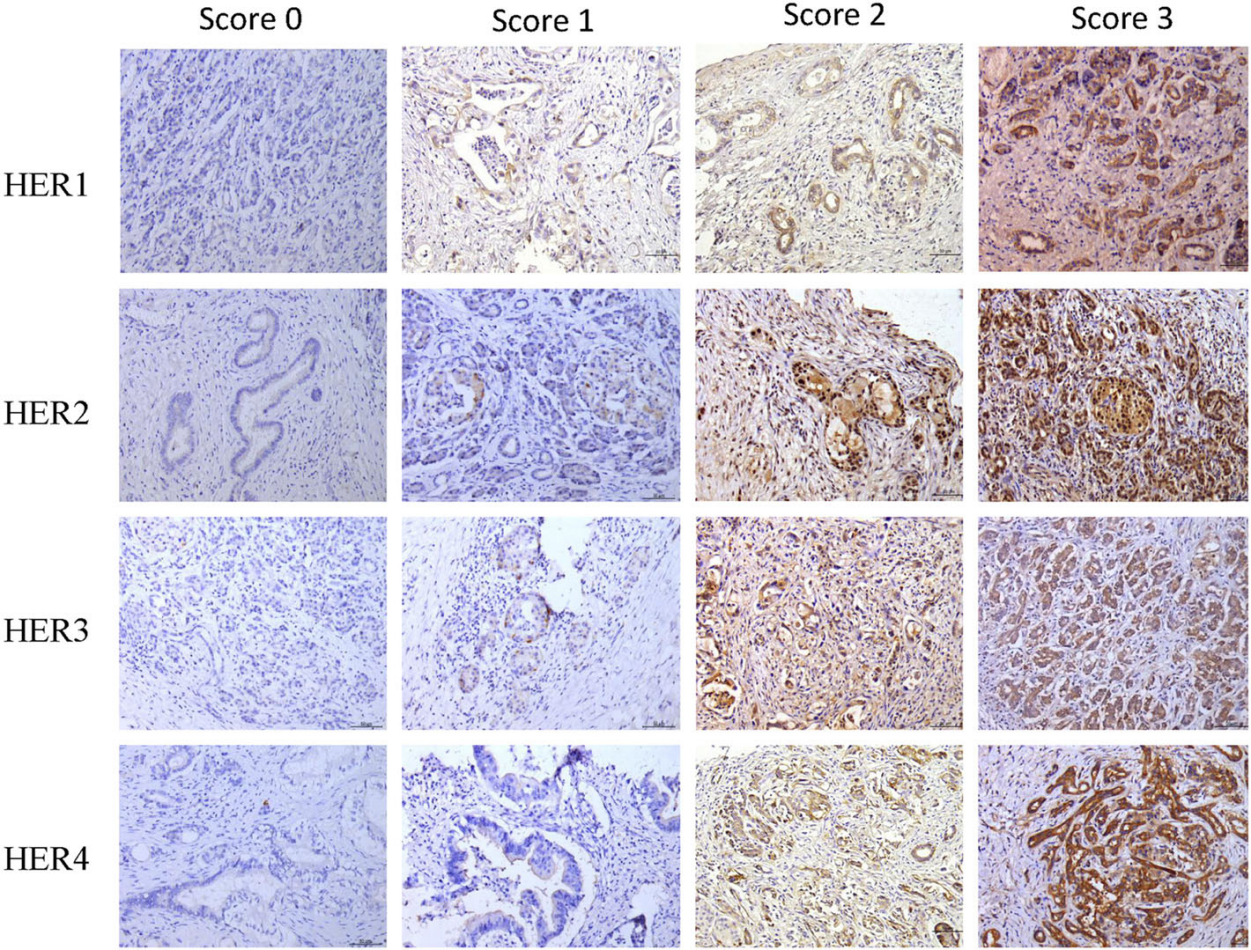
Representative images of HER1–4 tissue staining for all expression scores (0–3+) which were assigned according to the cytoplasm and membrane-staining intensity (original magnification ×200)

IHC analysis was performed to determine the expression of HER family proteins in tissue specimens obtained from 70 patients with operable pancreatic cancer. For each receptor, tissue specimens were assigned a score according to the cytoplasm and membrane-staining intensity. Analysis of intensity scores revealed that HER1, HER2, HER3 and HER4 exhibited positive staining in 41.4, 60.0, 24.3 and 65.7% of cases, respectively. Representative images of immunostaining related to HER1, 2, 3 & 4 expression in pancreatic cancer tissues are shown in Fig. 2.

### Analysis of the relationship between patient characteristics and median survival time in operable pancreatic cancer

The relationship between the clinical/pathological characteristics and median survival time in patients with operable pancreatic cancer are showed in Table 1. Differences in median survival times (MSTs) between subgroups were evaluated using the log-rank test. The MSTs of patients with stump cancer, low differentiation, adenocarcinoma, N1 stage, and CA 19-9 >1200U/ml were significantly lower than those of patients from the corresponding control subgroups (4.87 vs. 17.69 months, *P* < 0.001; 9.34 vs. 33.21 months, *P* < 0.001; 13.38 vs. 49.18 months, *P* < 0.001; 11.57 vs. 34.65 months, *P* = 0.007; 10.59 vs. 32.88 months, *P* < 0.001; respectively). No significant differences in MSTs were observed between subgroups of sex, age, smoking history, drinking history, tumor family history, tumor location, T stage, and M stage (Table 1).

**Table 1:** The comparison of median survival time in different subgroups

Variable	Number (%)	Median survival time (months)	Standard error (months)	*P* value
Sex				0.144
Male	28 (40.0)	11.93	3.98	
Female	42 (60.0)	16.64	6.11	
Age				0.059
< 60 y	34 (48.6)	49.18	16.69	
≥ 60 y	36 (51.4)	13.38	2.64	
Tumor family history				0.795
Yes	5 (7.1)	11.21	0.68	
No	65 (92.9)	16.64	4.38	
Smoking history				0.591
Yes	13 (18.6)	17.23	11.98	
No	57 (81.4)	15.88	4.18	
Drinking history				0.902
Yes	7 (10.0)	17.22	7.88	
No	63 (90.0)	15.88	4.41	
Tumor location				0.256
Head	45 (64.3)	13.38	1.92	
Body + tail	25 (35.7)	17.23	5.21	
Stump cancer				0.000
Yes	6 (8.6)	4.87	0.73	
No	64 (91.4)	17.69	8.53	
Differentiation				0.000
Lower	17 (24.3)	9.34	2.91	
Higher	53 (75.7)	33.21	18.47	
Pathology type				0.001
Adenocarcinoma	53 (75.7)	13.38	1.68	
Non-adenocarcinoma	17 (24.2)	49.18	1.02	
T stage				0.188
T1 + T2	34 (48.6)	22.32	11.19	
T3 + T4	36 (51.4)	12.07	2.20	
N stage				0.007
N0	39 (55.7)	34.65	13.76	
N1	31 (44.3)	11.57	0.79	
M stage				0.092
M0	62 (88.6)	17.23	5.86	
M1	8 (11.4)	7.66	4.67	
CA19-9				0.000
< 1200 U/ml	57 (81.4)	32.88	9.20	
> 1200 U/ml	13 (18.6)	10.59	3.29	

### HER family protein expression status among the different patient subgroups

The Chi-square test was used to analyze differences in HER family protein expression status between patient subgroups. Significant differences in HER1 expression status were observed between the sex subgroups (*P* = 0.027) and the differentiation subgroups (*P* = 0.026). A significant difference in HER2 expression status was observed between the CA 19-9 subgroups (*P* = 0.020). Significant differences in HER4 expression status were observed between the smoking history subgroups (*P* = 0.026), the drinking history subgroups (*P* = 0.042), the pathology types subgroups (*P* = 0.014) and the T stage subgroups (*P* = 0.017). However, significant differences in HER3 expression status were not observed between any subgroups (*P* > 0.05 in all cases; Table 2)

**Table 2:** The expression of HER1 - HER4 in different subgroups

	HER1			HER2			HER3			HER4		
	− *n*	+ *n*	*P*	− *n*	+ *n*	*P*	− *n*	+ *n*	*P*	− *n*	+ *n*	*P*
Sex			0.027			0.258			0.564			0.164
Male	12	16		13	15		21	7		12	16	
Female	29	13		15	27		32	10		12	30	
Age			0.663			0.519			0.446			0.139
< 60 y	19	15		14	20		25	9		9	25	
≥ 60 y	22	14		14	22		28	8		15	21	
Tumor family history			0.338			0.671			0.237			0.563
Yes	2	3		2	3		5	0		2	3	
No	39	26		26	39		48	17		22	43	
Smoking history			0.094			0.579			0.389			0.026
Yes	5	8		5	8		9	4		8	5	
No	36	21		23	34		44	13		16	41	
Drinking history			0.310			0.085			0.220			0.042
Yes	13	4		5	2		4	3		5	2	
No	28	25		23	40		49	14		19	44	
Tumor location			0.856			0.127			0.589			0.409
Head	26	19		15	30		35	10		17	28	
Body + tail	15	10		13	12		18	7		7	18	
Pathology types			0.138			0.233			0.113			0.014
Adenocarcinoma	28	24		19	33		37	15		22	30	
Non-adenocarcinoma	13	5		9	9		16	2		2	16	
Differentiation			0.026			0.167			0.144			0.570
Lower	6	11		9	8		15	2		6	11	
Higher	35	18		19	34		38	15		18	35	
T stage			0.663			0.330			0.164			0.017
T1 + T2	19	15		15	19		28	6		7	27	
T3 + T4	22	14		13	23		25	11		17	19	
N stage			0.566			0.480			0.496			0.329
N0	23	16		15	24		29	10		12	27	
N1	18	13		13	18		24	7		12	19	
M stage			0.273			0.598			0.091			0.269
M0	35	27		25	37		49	13		20	42	
M1	6	2		3	5		4	4		4	4	
CA199			0.467			0.020			0.332			0.480
< 1200 U/ml	34	23		19	38		42	15		19	38	
> 1200 U/ml	7	6		9	4		11	2		5	8	

### Association between HER family protein expression status and patient survival

**Fig. 3:**
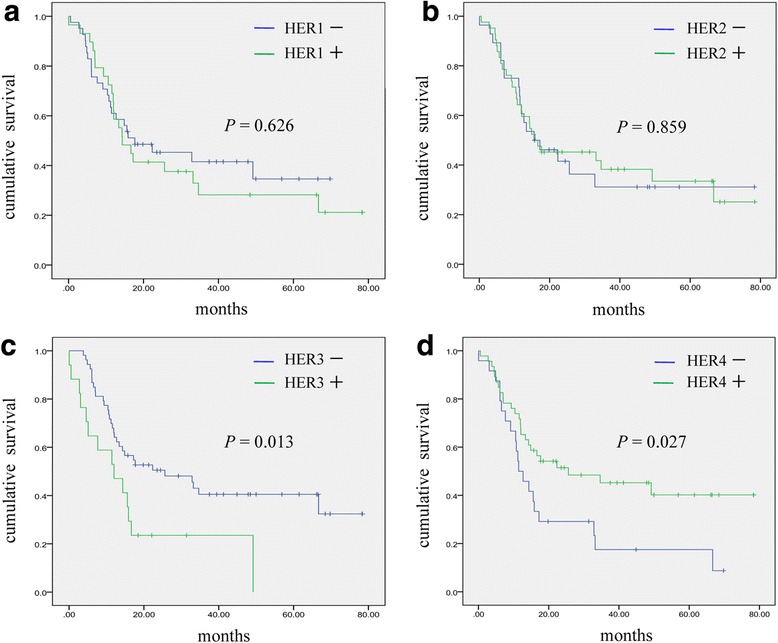
Kaplan–Meier survival curve analysis. Patients were categorized according to HER positive and negative expression status. Curves show overall survival differences according to HER1 (**a**), HER2 (**b**), HER3 (**c**), and HER4 (**d**) expression status

IHC analysis of patient tissue specimens revealed that positive HER1, HER2, HER3 and HER4 expression was observed in 41.4, 60.0, 24.3 and 65.7% of cases, respectively. Differences in patient survival times between the negative and positive expression subgroups for all HER family proteins were analyzed by the Kaplan–Meier method. The analysis showed that the MST for the HER3 positive group was 12.0 months, which was significantly lower than 25.6 months observed for the HER3 negative group (*P* = 0.013). MST for the HER4 positive group was 25.6 months, which was significantly higher than 11.4 months observed for the HER4 negative group (*P* = 0.027). No significant association between HER1 or HER2 expression status and survival time was observed in our analysis (*P* = 0.626 & *P* = 0.859, respectively; Fig. 3 and Table 3)

**Table 3:** The cancer-specific survival and HER1 - HER4 expression

Variable	Number (%)	Mean value (months)	Median survival time (months)	*P* value (for median survival time)
HER1				0.626
Negative	41 (58.6)	34.0	17.7	
Positive	29 (41.4)	31.4	14.3	
HER2				0.859
Negative	28 (40.0)	33.4	15.6	
Positive	42 (60.0)	35.1	15.9	
HER3				0.013
Negative	53 (75.7)	39.1	25.6	
Positive	17 (24.3)	18.1	12.0	
HER4				0.027
Negative	24 (34.3)	24.5	11.4	
Positive	46 (65.7)	40.6	25.6	

### Evaluation of HER family protein expression status and clinicopathological characteristics as independent predictors of survival in operable pancreatic cancer

A large number of factors affected the survival time of patients with operable pancreatic cancer, and Cox’s multiple regression analysis was performed to identify those factors that were independent predictors of prognosis. HER family protein expression status along with those clinicopathological parameters which demonstrated significance following univariate analysis were included in the Cox’s multiple regression analysis. Risk of death for the HER3 positive group was 3.684 times greater than that for the HER3 negative group (*P* = 0.001). Differentiation (RR 3.804, *P* = 0.006), histopathology type (RR 4.595, *P* = 0.013) and stump cancer (RR 10.110, *P* = 0.001) were also independent predictors of survival. However, the expression status of HER1, HER2 and HER4, positive lymph node number, N stage and CA 19-9 score could not independently predict survival (*P* > 0.05 in all cases; Table 4).

**Table 4:** Cox’s multiple regression analysis for survival time

Variable	Relative risk	95% confidence interval	*P* value
HER1 positive vs. negative	0.598	0.281–1.271	0.181
HER2 positive vs. negative	0.909	0.420–1.968	0.809
HER3 positive vs. negative	3.684	1.732–7.834	0.001
HER4 positive vs. negative	0.800	0.375–1.710	0.565
Stump cancer yes vs. no	10.110	2.725–37.506	0.001
Histopathology type Adenocarcinoma vs. non-adenocarcinoma	4.595	1.371–15.404	0.013
Differentiation poor vs. well	3.804	1.480–9.774	0.006
N stage N1 vs. N0	0.661	0.259–1.685	0.386
The positive lymph node number	1.331	0.964–1.838	0.083
CA199 >1200 U/ml vs. <1200 U/ml	1.961	0.806–4.770	0.138

## Discussion

There are relatively few studies on the prognostic significance of HER family members in patients with operable pancreatic cancer. To date, the findings regarding the prognostic significance of HER family protein expression in pancreatic cancer have been inconsistent. This may be a consequence of inter-study differences in tissue preparation, the detection antibodies used, and different methods of scoring. In our study we performed a comprehensive analysis of the prognostic value of HER1–4 in patients with operable pancreatic cancer. Kaplan–Meier analysis showed that the MST of the HER3 positive group was 12.0 months, which was significantly lower than 25.6 months observed for the HER3 negative group (*P* = 0.013). Cox’s multiple regression analysis demonstrated that HER3 was an independent predictor of poor prognosis; risk of death associated with HER3 positive expression was 3.684 times greater than that associated with HER3 negative expression (*P* = 0.001). Although Cox’s multiple regression analysis did not identify HER4 positive expression as an independent predictor of survival, Kaplan–Meier analysis did show that the MST of the HER4 positive group was 25.6 months, which was significantly higher than 11.4 months for the HER4 negative group (*P* = 0.027). This suggests that HER4 may be of some potential prognostic value in pancreatic cancer and this deserves further attention. No significant association between HER1 or HER2 positive expression and survival were observed, and neither receptor was a predictor of survival.

HER1 is overexpressed in a variety of human malignancies [21, 22]. The frequency of HER1 expression in pancreatic cancer has been reported as 30.4% [23] and 45.1% [25] in two previous studies. In our study HER1 expression was observed in 41.4% of patients. HER1 overexpression has previously been associated with decreased survival [23, 24], and the cumulative 1-, 3- and 5-year survival rates were 48, 20 and 18%, respectively [24]. A previous meta-analysis has shown that three trials reported a survival disadvantage for patients with HER1 expression, while other five trials reported no significant difference, however, the combined hazard ratio was 1.225 (*P* = 0.035) [25]. Another meta-analysis drew the opposite conclusion that HER1 was not a significant prognostic factor in resected pancreatic cancer (HR = 1.35, 95% CI 0.80–2.27, *P* > 0.05) [26]. From our analysis, we also drew the conclusion that positive HER1 expression has no prognostic value in operable pancreatic cancer.

HER2 expression is associated with decreased disease-specific survival and poor prognosis in patients with breast cancer and gastric cancer [17, 18, 27]. The frequency of HER2 expression and its association with survival in pancreatic cancer remains unclear. Te Velde et al. reported that neither membranous overexpression nor gene amplification of HER2 was seen in pancreatic cancer [28]. A study by Aumayr, et al. observed positive HER2 expression, as determined by IHC staining, in 10% of pancreatic cancer cases, and of these, 7% demonstrated HER2 gene amplification [29]. In our study we observed positive HER2 expression in 60.0% of cases, which is significantly higher than that observed by others. Komoto et al. reported that patients with HER2 overexpression had significantly shorter survival times than those with normal HER2 expression (14.7 vs. 20.7 months, *P* = 0.008), and multivariate survival analysis demonstrated that HER2 was an independent prognostic factor (HR 1.806; *P* = 0.026) [30]. In our study, however, no significant association between HER2 expression and survival time was observed, and there was no prognostic value associated with positive HER2 expression.

Our study has confirmed the high expression of pHER3 in the AsPC-1 pancreatic cancer cell line by western blotting, and anti-HER3 monoclonal antibody could significantly down-regulated pHER3 level. Furthermore, the treatment of AsPC-1 cells with increasing concentrations of anti-HER3 monoclonal antibody resulted in significant inhibition of cellular proliferation. HER2/HER3 heterodimers are reported to be activated in pancreatic cancer [31], the fact that anti-HER3 monoclonal antibody (3D4) and anti-HER2 monoclonal antibody (trastuzumab) synergetic down-regulated pHER3 in the AsPC-1 cell indirectly proved the simultaneous inactivation of HER2/HER3 heterodimers. These results of Thomas G et al. and our study are identical. However, the diverse coexistence of HER family homo or heterodimers (HER2/HER2, HER1/HER2, HER1/HER3 and HER2/HER3) and differences in detection methods may explain the non-parallel expression of HER2 and HER3. Studies on the predictive value of HER3 expression on patient outcome in solid tumors are limited. A meta-analysis has reported HER3 overexpression in 42.2% of solid tumors [32] and an association between HER3 expression and worse overall survival at both 3 years (OR = 2.24, *P* < 0.001) and 5 years (OR = 2.20, *P* < 0.001) [32]. Thomas et al. reported that HER3 was expressed in 27% of the 45 cases of pancreatic ductal adenocarcinoma, which was similar to the 24.3% reported in our study [31]. The study by Thomas et al. showed that HER3 expression was essential for pertuzumab efficacy in HER2low pancreatic cancer, and that HER3 expression may be a predictive biomarker for pertuzumab efficacy [31]. Hirakawa at al. reported that the MST for patients with curatively resected pancreatic cancer associated with HER3 overexpression was 37.2 months, while that for HER3-negative patients was 58.6 months (*P* = 0.008) [20], the findings was similar to those of our study; our Cox’s regression analysis demonstrated that the risk of death associated with HER3 positive expression was 3.684 fold greater than that associated with HER3 negative expression. We therefore conclude that HER3 is a novel independent prognostic biomarker for poor survival in patients with pancreatic cancer.

Clinical research reports on the significance of HER4 expression in the prognosis of patients with solid tumors are less numerous than for other HER family members. It has been reported that the level of HER4 in pancreatic cancer specimens, as determined by IHC, has no association with patient survival [33]. A study by Thybusch-Bernhardt et al. suggested that HER1 and HER2 overexpression contributes to a more aggressive phenotype and that lack of HER4 expression may increase the metastatic capacity of pancreatic cancer cells [34]. This is consistent with our finding that the MST for the HER4 positive patient group was significantly higher than that for the HER4 negative group (25.6 vs. 11.4 months, *P* = 0.027). Moreover, HER4 expression was negatively associated with adenocarcinoma, which was shown to be a poor outcome indicator in our study. However, Cox’s regression analysis did not identify HER4 as an independent predictor of survival.

## Conclusions

In conclusion, our study shows that HER3 is a novel independent prognostic marker for patients with operable pancreatic cancer. There is a potentially positive advantage associated with HER4 expression which deserves further attention. The prognostic significance associated with HER1 and HER2 expression in operable pancreatic cancer was not found. By analyzing the expression of all HER family members together within a single study enable us to ensure consistency in specimen processing, detection, and scoring methods. This approach enables more robust conclusions to be drawn when comparing the prognostic significance of the single HER family proteins. However, our study is limited by the small number of cases, and a larger cohort prospective study is necessary to confirm our conclusions. The interactions among ligands, HER family members and downstream signaling molecules are intricate, and the patterns of coexpression of the signaling molecules will need to be taken into account to fully understand the prognostic significance within a given disease context. Advances in detection technology and standardization of diagnosis criteria will continue to improve methods for determining the prognostic value of HER family members in operable pancreatic cancer.

## Abbreviations

HER: Human epidermal growth factor receptor
IHC: Immunohistochemistry
MST: Median survival times
